# Molecularly imprinted MOF nanozymes: Demonstration of smartphone-integrated dual-mode platform for ratiometric fluorescent/colorimetric detection of chloramphenicol

**DOI:** 10.1016/j.fochx.2025.102322

**Published:** 2025-02-25

**Authors:** Xiang-Yi He, Ya Wang, Qin Xue, Wan-Fen Qian, Guang-Li Li, Qing Li

**Affiliations:** a College of Life Science asnd Chemistry, Hunan University of Technology, Zhuzhou 412007, China; b Zhuzhou Institute for Food and Drug Control, Zhuzhou 412007, China

**Keywords:** Ratiometric fluorescent sensor, Colorimetric analysis, Dual-mode, Molecularly imprinted nanozyme, Chloramphenicol

## Abstract

Reliable and accurate determination of chloramphenicol (CAP) is urgently needed due to its significant implications for food safety and human health concerns. In this study, we successfully synthesized MIL-101(Fe)-NH_2_@MIP, which exhibits peroxidase activity and a specific recognition function for CAP, through in situ polymerization of dopamine on the surface of MIL-101(Fe)-NH_2_, utilizing molecular imprinting technology. Our bifunctional MIL-101(Fe)-NH_2_@MIP probe offers a ratiometric fluorescent and colorimetric dual-mode sensing strategy for sensitive and selective detection of chloramphenicol. The detection limits for CAP can reach down to 36.45 nM and 93.38 nM, respectively. Furthermore, we developed a smartphone-based visual sensing platform that employs MIL-101(Fe)-NH_2_@MIP nanozymes for rapid, portable, low-cost on-site detection of CAP. Successful spike recovery experiments conducted in fresh milk samples further validate the potential practical application of our proposed dual-mode strategy. Thus, this approach expands the applications of MOFs-based nanozymes and holds promise for antibiotic determination.

## Highlights

• A MIL-101(Fe)-NH_2_@MIP sensing platform for chloramphenicol is reported.
• A novel dual-mode sensing platform can be established.
• The favorable selectivity and sensitivity were achieved.
• This strategy enables on-site detection of chloramphenicol.

## 1 Introduction

Chloramphenicol (CAP) is a broad-spectrum antibiotic that exhibits inhibitory effects on the growth of pathogenic Gram-positive and Gram-negative bacteria. It functions by inhibiting protein synthesis in bacteria, ultimately leading to their death. CAP has been widely utilized in both animal husbandry and clinical settings (Pan & Chu, 2017). However, residues of CAP can enter the human body through animal products or environmental exposure, resulting in adverse health effects such as aplastic anemia and myelosuppression, severe cases may even lead to cardiovascular collapse syndrome (Darabi et al., 2021). Currently, countries including China and the United States have enacted policies prohibiting the use of CAP in all animal feed products. Nevertheless, due to its low cost and high efficacy, illegal usage of CAP continues within aquaculture practices. Reports indicate that chloramphenicol residues have been detected in water bodies such as Dong hu Lake, the Pearl River, and the Yangtze River in China (Li et al., 2020). Therefore, monitoring CAP concentrations is essential for guaranteeing food safety and protecting human health.

Diverse analytical strategies for detecting CAP have been developed, including enzyme-linked immunosorbent assay (ELISA) (Guo et al., 2023), high-performance liquid chromatography (HPLC) (Mehrabi & Ghaedi, 2023), gas chromatography (GC) (Guidi et al., 2017), and liquid chromatography-tandem mass spectrometry (LC-MS/MS) (Sun et al., 2017). Although HPLC, GC, and LC-MS/MS are highly sensitive techniques, they possess several drawbacks of high cost, the need for specialized technicians as well as sophisticated instrumentation, complex operation and time-consuming sample pre-treatment, which restrict their implementation in field-based rapid screening processes. Conversely, while ELISA offers fast detection speeds and straightforward operation protocols, it suffers from lower sensitivity levels and a propensity for false negative results (Huang et al., 2020; Luo et al., 2018). Consequently, an immediate demand exists for a rapid, simple and cost-effective approach for on-site detection of chloramphenicol.

Optical biosensors offer a simpler development process compared to other analytical methods, primarily due to their rapid construction, high performance, and exceptional sensitivity (Guo et al., 2024). The widespread availability of smartphones allows for the easy conversion of optical signals into RGB values, facilitating the quantitative assessment of targets using color recognition applications (Qiu et al., 2017; Yu et al., 2021). This feature significantly enhances the practical application of optical biosensors for on-site and rapid analyses (Chen et al., 2025). Recently, a variety of optical sensors have been developed for analysis of CAP (Zhao et al., 2023; Zou et al., 2025). However, it is important to note that these optical sensors are primarily designed with single-mode and single-signal output configurations for CAP detection. Consequently, they exhibit limited resistance to interfering agents, which may lead to skewed data. In contrast to single-signal sensors, multi-signal ratiometric optical sensors include integrated self-calibration features that efficiently reduce the impact of diverse extraneous factors influencing experiment outcomes (Lai et al., 2024; Wu et al., 2023). This allows for accurate detection of target molecules within complex matrices. Unlike multi-signal ratiometric optical sensors that produce identical types of signal molecules, multi-mode optical sensors can generate diverse categories of signal outputs (Li, Guo, et al., 2023; Li, Qi, et al., 2023). The output signals from these sensors are independent and do not interfere with one another, simultaneously, each output signal can validate the others. This feature helps prevent interfering agent present in complicated sample matrices from compromising experimental outcomes-thereby enhancing both the authenticity and reliability of detection results (Qiu et al., 2018; Wu et al., 2024). In recent years, several studies have integrated colorimetry with fluorescence techniques to develop dual-mode sensing platforms, these platforms take advantage of the straightforward and rapid detection advantages inherent in colorimetry while incorporating a fluorescence detection method characterized by higher sensitivity (Zhang et al., 2024; Zheng et al., 2024). Such integration broadens the applicability of this detection approach and facilitates mutual confirmation between results obtained from both modes. However, a considerable number of colorimetric and fluorescent detection platforms necessitate the sequential analysis of multiple substances. Alternatively, it may be impractical to utilize both detection methods simultaneously under the same experimental conditions.

Metal-organic frameworks (MOFs) are formed from metal ions and organic linkers through robust covalent bonds (Yuan et al., 2018). The structural characteristics of MOFs provide numerous opportunities for designing fluorometric sensing approaches, as both organic linkers and metal ions can contribute to the generation of fluorescence signals (Jie et al., 2023). Furthermore, A variety of MOFs incorporating active metal redox pairs have been demonstrated to exhibit enzyme-like functions that catalyze substrates reactions (Wang et al., 2023). Additionally, MOFs exhibit enzyme-mimicking properties that demonstrate greater stability in harsh conditions-such as harsh pH levels, high salinity, elevated temperatures, and prolonged storage-compared to natural enzymes (Yu et al., 2023; Yu et al., 2024). These capabilities can improve analytical sensitivity through signal amplification resulting from catalytic reactions. Notably, these desirable features can be effectively incorporated into a framework to create a composite probe for targets detection, significantly broadening the applications possibilities of MOFs. Molecular imprinting represents a significant bio-similarity recognition technology, which is designed to imitate the specific recognition capabilities of antibodies and enzymes. In contrast to biological recognition, molecular imprinting polymer (MIP) demonstrate considerable selectivity and specificity toward the target analytes. More importantly, they also offer advantages such as enhanced stability, straightforward preparation processes, and cost-effectiveness (Miao et al., 2024). Therefore, the combination of a multifunctional composite MOF probe with molecular imprinting technology to develop an integrated multi-mode sensor holds promising applications in the field of biochemical analysis.

**Scheme 1:**
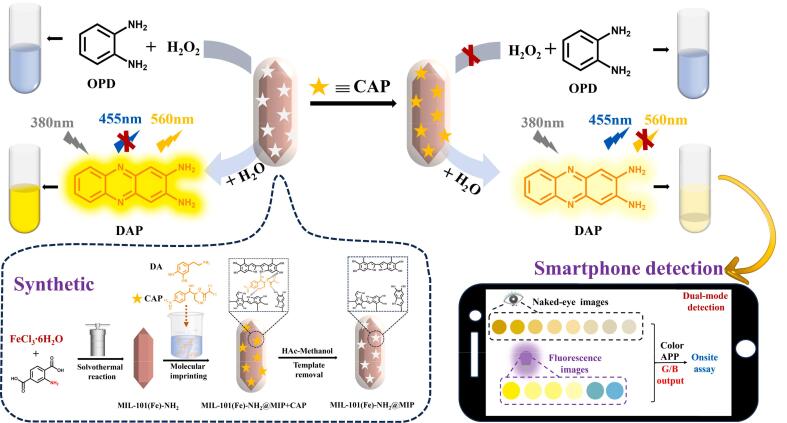
Schematic representation of colorimetric/ratiometric fluorescent dual-mode platform determination of chloramphenicol via MIL-101 (Fe)-NH_2_@MIP.

Herein, we combined a difunctional iron-based metal-organic framework (MOF) with MIP to develop a ratiometric fluorescent and colorimetric dual-mode sensor for determination of chloramphenicol (CAP). The sensing principle is illustrated in Scheme 1. The bifunctional MIL-101(Fe)-NH_2_ was synthesized via solvothermal process using FeCl_3_·6H_2_O as the metal node and NH_2_-BDC as the organic ligand. The use of NH_2_-BDC incorporates an emission peak at 455 nm in MIL-101(Fe)-NH_2_ when excited at 380 nm, while the mixed valence metal node of Fe^3+^/Fe^2+^ contributes significantly to the material's peroxidase-like activity. This activity enables the oxidation of colorless *o*-phenylenediamine (OPD) to form a yellowish solution of 2,3-diaminophenazine (DAP) in the presence of H_2_O_2_. Notably, DAP exhibits a prominent signal at 560 nm when excited at 455 nm, which suppresses the intrinsic peak of fluorescent MIL-101(Fe)-NH_2_ at 455 nm due to the inner filter effect (IFE). Subsequently, we prepared the core-shell structure MIL-101(Fe)-NH_2_@MIP, which specifically recognizes CAP, through straightforward self-polymerization of dopamine (DA) under gentle conditions. In this process, DA served as the functional monomer while CAP acted as the template molecule. MIL-101(Fe)-NH_2_@MIP has been demonstrated to have fluorescence properties and peroxidase-mimic activity similar to those of MIL-101(Fe)-NH_2_. Upon the complete elution of CAP, numerous cavities are exposed on MIL-101(Fe)-NH_2_@MIP. In this situation, hydrogen peroxide (H_2_O_2_) can smoothly enter MIL-101(Fe)-NH_2_@MIP through its channels and stimulate its peroxidase-like activity to generate hydroxyl radicals (·OH). This subsequently oxidizes OPD to form yellowish DAP. In the meantime, the fluorescence of MIL-101(Fe)-NH_2_@MIP at 455 nm decreases and the fluorescence of DAP at 560 nm increases upon 380 nm irradiation. When CAP is present, it becomes trapped by the MIP shell and binds specifically to recognized cavities on MIL-101(Fe)-NH_2_@MIP. This binding partially obstructs H_2_O_2_ entry into both channels and cavities, thereby inhibiting hydroxyl radical production. As a result, catalyzed OPD chromogenic reactions are hindered. Simultaneously, the fluorescence signal at 560 nm from DAP is suppressed, while the intrinsic peak fluorescence signal at 455 nm is restored. Based on the changes of fluorescence intensity ratio F_560_/F_455_ (where F_560_ and F_455_ denote the ratios of fluorescence intensities value at 560 nm and 455 nm) and the chromogenic properties, a highly selective ratiometric fluorescence/colorimetric dual-mode quantitative analysis of CAP concentration was validated. Furthermore, a smartphone sensing platform was developed for detection of CAP by converting the results into digital color RGB analysis using simple color selector software.

## 2 Experimental section

### 2.1 Material and apparatus

*N*, *N*-dimethylformamide (DMF), 2-Aminoterephthalic acid (NH_2_-BDC), FeCl_3_·6H_2_O, dopamine hydrochloride, chloramphenicol (CAP), and *o*-phenylenediamine (OPD), 3,3′,5,5′-tetramethylbenzidine (TMB) were supplied by Aladdin Reagent Co. Ltd. (Shanghai, China). H_2_O_2_ (30 %), ethanol, methanol, anhydrous sodium acetate (CH_3_COONa), and acetic acid (CH_3_COOH) were ordered from Sinopharm Chemical Reagent Co. (Shanghai, China).

Fluorescence spectra were obtained using a Hitachi F-7100 fluorescence spectrometer (Tokyo, Japan), configured with an excitation slit width of 10 nm, an emission slit width of 10 nm, and a photomultiplier voltage set at 700 V. Absorption spectra were recorded on a Lambda 750 s UV–Vis spectrometer from PerkinElmer (USA).

### 2.2 Preparation of MIL-101(Fe)-NH_2_ and MIL-101(Fe)-NH_2_@MIP

The synthesis of MIL-101(Fe)-NH_2_ was conducted via a straightforward solvothermal method (Chu et al., 2022). Solution A was prepared by dissolving 2.5 mM FeCl_3_·6H_2_O in 15 mL of DMF, while solution B was formed by dissolving 2.5 mM NH_2_-BDC in 10 mL of DMF. Subsequently, solutions A and B were combined and transferred to a 50 mL autoclave where they reacted at 120 °C for 12 h. After cooling to room temperature, the product was harvested via centrifugation and rinsed three times with deionized water and ethanol to discard any residual metal ions and ligands. Consequently, the resulting solid was dried at 60 °C to obtain MIL-101(Fe)-NH_2_.

MIL-101(Fe)-NH_2_@MIP composites were prepared following established literature protocols (Zhang et al., 2021). The preparation involved self-polymerization of CAP under mild conditions: MIL-101(Fe)-NH_2_ (40 mg) was dispersed in 60 mL of 10 mM Tris-HCl buffer (pH 8.0), sonicated for 10 min, and magnetically stirred for 20 min at room temperature until uniform dispersion occurred. Following this step, CAP (20 mg) was added and continuously stirred under moderate magnetic force for two hours. Subsequently, dopamine hydrochloride (80 mg) was introduced at room temperature with continuous mechanical stirring in darkness for five hours. The resulting solid product underwent washing with Acetic acid - methanol (1:9 *v*/v) to remove the template molecule CAP until no UV–visible absorption at 280 nm could be detected. Ultimately, MIL-101(Fe)-NH_2_@MIP underwent vacuum drying. The nonimprinted polymer MIL-101(Fe)-NH_2_@NIP was synthesized similarly. However, it did not involve the addition of the template molecule (CAP).

### 2.3 Peroxidase-mimic activities of MIL-101(Fe)-NH_2_ and MIL-101(Fe)-NH_2_@MIP

The kinetic analysis of MIL-101(Fe)-NH_2_ and MIL-101(Fe)-NH_2_@MIP were determined by using TMB as substrate. The experimental procedure is presented as follows: a series of various concentrations of 10 μL TMB solutions, 10 μL MIL-101(Fe)-NH_2_ or MIL-101(Fe)-NH_2_@MIP (0.1 mg/mL), 10 μL H_2_O_2_ (1 M) and NaAc-HAc buffer (pH = 4.5, 0.2 M) were mixed and diluted to 100 μL with ultrapure water. The absorption spectrum of the mixture after incubated 30 min was recorded.

### 2.4 Colorimetric and ratiometric fluorescent sensing of CAP

A total of 10 μL of MIL-101(Fe)-NH_2_@MIP suspension (0.1 mg/mL), along with 10 μL of various concentrations of CAP solution, was dropped to a centrifuge tube including NaAc-HAc buffer (pH = 4.5, 0.2 M). The mixture was incubated for 20 min. Subsequently, 10 μL of a 1 M H_2_O_2_ solution and 10 μL of 10 mM OPD solution were introduced into this mixture. The catalytic reaction proceeded for an additional 30 min at room temperature before UV–vis absorption spectra and fluorescence emission spectrum were recorded. The fluorescence signals output value was determined based on the ratio of fluorescence intensity (F_560_/F_455_), where F_560_ and F_455_ denote the ratios of fluorescence intensities value at 560 nm and 455 nm.

### 2.5 Detection of CAP in practical samples

To assess the feasibility of this developed method for detecting CAP in actual samples, the fresh milk samples were bought from a local supermarket. The treatment method for the milk samples was adapted from previous reports (Xu et al., 2021). 4 mL of fresh milk samples was mixed with 1 mL of TCA (1 %). The mixture was stirred for 10 min, followed by the addition of 0.5 mL of methanol and thorough stirring. Ultrasonic treatment was then applied for 20 min, after which centrifugation at 10,000 rpm for 10 min was performed to remove organic substances such as proteins and lipids. The resulting supernatant was filtered using a 0.22 μm filter membrane. Subsequently, known concentrations of CAP were then added to these prepared samples prior to detection using procedures similar to those described above.

## 3 Results and discussion

### 3.1 Characterization of MIL-101(Fe)-NH_2_ and MIL-101(Fe)-NH_2_@MIP

**Fig. 1:**
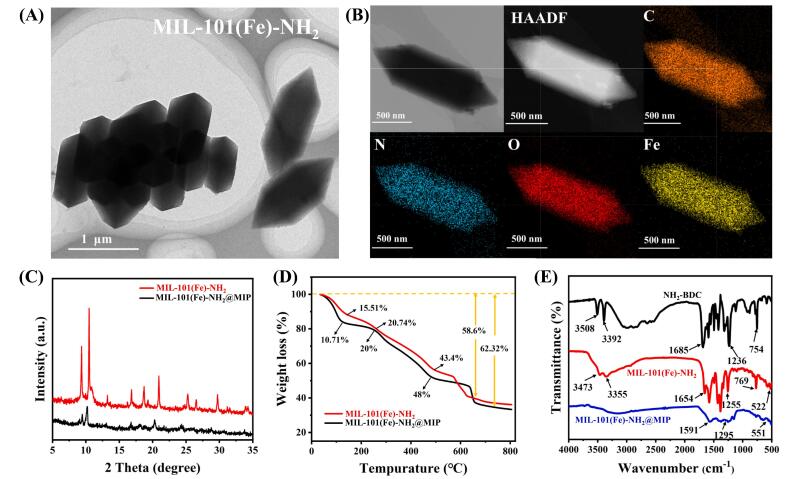
(A) TEM image and EDS elemental mapping; (B)of MIL-101(Fe)-NH_2_; (C) The XRD patterns and TGA curves; (D) of MIL-101(Fe)-NH_2_ and MIL-101(Fe)-NH_2_@MIP; (E) The FTIR spectrum of NH_2_-BDC, MIL-101 (Fe)-NH_2_ and MIL-101 (Fe)-NH_2_@MIP.

The structures and morphologies of both MIL-101(Fe)-NH_2_ and MIL-101(Fe)-NH_2_@MIP underwent systematic characterization. As illustrated in Fig. 1A, transmission electron microscopy (TEM) revealed that MIL-101(Fe)-NH_2_ exhibited uniform octahedral shapes. Furthermore, elemental mapping (Fig. 1B) indicated that C, O, N, and Fe elements were uniformly distributed within it. In addition, the presence of C, O, N and Fe elements in MIL-101(Fe)-NH_2_ was validated by energy spectrum analysis (EDS) (Fig. S1). During the synthesis process, MIL-101(Fe)-NH_2_@MIP incorporated template molecules, which can be thoroughly eluted. As illustrated in Fig. S2, after washing MIL-101(Fe)-NH_2_@MIP eight times, the supernatant exhibited no discernible UV absorption feature at 280 nm associated with CAP. It is evident from Fig. S3 that an imprinted layer constructed on the surface of MIL-101(Fe)-NH_2_, resulting in a rough and irregular surface texture. X-ray diffraction (XRD) analysis (Fig. 1C) was employed to characterize both synthesized MIL-101(Fe)-NH_2_ and MIL-101(Fe)-NH_2_@MIP. The diffraction peaks of MIL-101(Fe)-NH_2_ observed at 2θ = 9.28^o^, 10.42^o^, 13.16^o^, 16.8^o^, 18.61^o^ were consistent with those reported in the literature (Chu et al., 2022), indicating good crystallinity. Notably, MIL-101(Fe)-NH_2_@MIP displayed identical diffraction peak as those of MIL-101(Fe)-NH_2_. However, their intensity was slightly reduced because of the MIPs membrane coverage.

Thermogravimetric analysis (TGA) confirmed the presence of an imprinting layer on the surface of MIL-101(Fe)-NH_2_ (Fig. 1D). As the temperature increases, both MIL-101(Fe)-NH_2_ and MIL-101(Fe)-NH_2_@MIP display three distinct stages of weight loss from room temperature to approximately 125 °C. The initial weight loss, accounting for 15.51 % and 10.7 %, respectively, is ascribed to the evaporation of absorbed water. The second stage, characterized by a weight loss of 20.74 % and 20 %, occurs between approximately 125 °C and 255 °C. This is due to the elimination of carboxyl groups as well as the volatilization of unreacted reactants that have not fully participated in the reaction process (Liu et al., 2022). The third stage involves weight loss occurring between 255 °C and 490 °C, with values recorded at 43.4 % and 48 %, respectively. During this phase, coordination bonds within MIL-101(Fe)-NH_2_ begin to break down (Luo & Su, 2024). Beyond temperatures exceeding 490 °C, a complete collapse of the metal-organic framework is observed. Notably, in this final step, the percentage weight loss for MIL-101(Fe)-NH_2_@MIP was found to be higher at 62.32 % compared to that of MIL-101(Fe)-NH_2_, which was measured at 58.6 %. The results showed that PDA formed a coating layer on MIL-101(Fe)-NH_2_ surface. PDA is a zwitterionic polymer that includes amines and phenolic hydroxyl groups. In neutral or alkaline conditions, deprotonation of the phenol hydroxyl group lead to a negative charge (Shen et al., 2024) This change is reflected in Zeta potential measurements showing a negative Zeta potential following PDA capping (Fig. S4).

Fourier transform infrared (FTIR) spectroscopy presented in Fig. 1E, reveals that the C=O stretching vibration absorption peak for NH_2_-BDC appeared at 1685 cm^−1^ but shifted to 1654 cm^−1^ upon formation of MIL-101(Fe)-NH_2_. This shift confirms coordination interaction between Fe ions and -COOH on NH_2_-BDC occurred during synthesis. Additionally, double peaks observed at 3508 cm^−1^ and 3392 cm^−1^ correspond to symmetric and asymmetric tensile vibrations of -NH_2_ groups within NH_2_-BDC when compared to peaks at 3473 cm^−1^ and 3355 cm^−1^ found in MIL-101(Fe)-NH_2_. These findings indicate that amino group are indeed present within the structure of MIL-101(Fe)-NH_2_. The peak at 1236 cm^−1^ corresponds to the stretching vibration of C—N in NH_2_-BDC, which is shifted to 1255 cm^−1^ in MIL-101(Fe)-NH_2_. Additionally, a metal‑oxygen bond peak was identified at 522 cm^−1^, confirming the generation of Fe—O bond. The C—H bending vibration peak on the benzene ring moved from 754 cm^−1^ to 769 cm^−1^ because of the interaction between -COOH and Fe^3+^. Furthermore, peaks at 1295 cm^−1^ in the FTIR spectrum of MIL-101(Fe)-NH_2_@MIP was attributed to the stretching vibration of C—N in polydopamine, while the characteristic peak of Fe—O bond shifted to 551 cm^−1^.

It is hypothesized that MIL-101(Fe)-NH_2_ exhibits photoluminescent attributed to its ligand. As shown in Fig. S5A, spectra indicate that the maximum excitation wavelength for MIL-101(Fe)-NH_2_ occurs at 350 nm, with a corresponding maximum emission wavelength at 455 nm. This aligns with observations made for NH_2_-BDC (Fig. S5B). These results suggest that the fluorescence properties of MIL-101(Fe)-NH_2_ are derived from its ligand. Moreover, fluorescence emissions under varying excitation wavelengths were surveyed (Fig. S5C). The fluorescence intensity increased gradually as excitation wavelengths increased from 340 nm to 350 nm but decreased within a range from 350 nm to 385 nm. Notably, the emission peak remained fixed at 455 nm regardless of excitation conditions. Similar behavior was observed for MIL-101(Fe)-NH_2_@MIP (Fig. S6A), which also exhibited maximum excitation and emission peaks at wavelengths of 350 nm and 455 nm, respectively. Additionally, their emission signals demonstrated similar independence concerning excitation across a range from approximately 340–385 nm (Fig. S6B). These results suggest that coating with an imprinted layer has minimal impact on the intrinsic fluorescence properties of this metal-organic framework (MOF). Importantly, it should be noted that MIL-101(Fe)-NH_2_@MIP also exhibit excellent photostability and chemical stability (Figs. S7 and S8). Collectively, these findings further verified the successful preparation of both MIL-101(Fe)-NH_2_ and MIL-101(Fe)-NH_2_@MIP.

### 3.2 Investigation of the peroxidase-mimic activity of MIL-101(Fe)-NH_2_@MIP

**Fig. 2:**
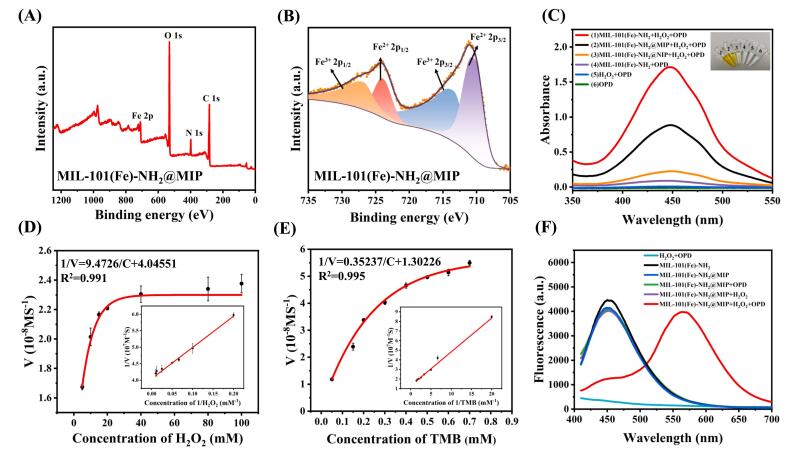
XPS spectrum (A) of MIL-101(Fe)-NH_2_@MIP and high-resolution XPS spectrum (B) of Fe 2p; (C) UV–vis spectra of different systems; (D) and (E) Steady-state kinetics analysis of MIL-101(Fe)-NH₂@MIP; (F) Fluorescence spectra of different systems.

The chemical composition of the synthesized MIL-101(Fe)-NH_2_ and MIL-101(Fe)-NH_2_@MIP was further analyzed using X-ray photoelectron spectroscopy (XPS). As illustrated in Fig. S9A, the peaks attributed to the four elements — C 1 s, N 1 s, O 1 s and Fe 2p — are located at 284.1 eV, 398.9 eV, 531.1 eV and 711.4 eV, respectively. The high resolution XPS spectrum for Fe 2p revealed that both Fe^2+^ and Fe^3+^ were present in MIL-101(Fe)-NH_2_ (Fig. S9B). Specifically, the peaks at 726.45 eV and 715.14 eV are attributed to Fe^3+^, while those at 724.24 eV and 711.43 eV are attributed to Fe^2+^. The full XPS spectrum (Fig. 2A) also confirms the presence of Fe, O, C, and N elements within MIL-101(Fe)-NH_2_@MIP. In MIL-101(Fe)-NH_2_@MIP, the peaks observed at 726.79 eV and 713.45 eV are associated with Fe^3+^, whereas those at 723.93 eV and 710.38 eV correspond to Fe^2+^ (Fig. 2B). This indicated that mixed valence states of Fe^2+^/Fe^3+^ also existed in MIL-101(Fe)-NH_2_@MIP. The presence of these mixed valence states is crucial for facilitating oxidation-reduction reaction between H_2_O_2_ and TMB since they can initiate a Fenton-like reaction involving Fe^2+^/Fe^3+^ with H_2_O_2_. This leads to the generation of highly reactive oxidizing agents such as hydroxyl radicals which subsequently trigger additional reactions that enhance peroxidase-like activity in metal-organic frameworks.

The peroxidase-mimic activity of MIL-101(Fe)-NH_2_@MIP was investigated through catalytic oxidation using OPD as a chromogenic reagent in conjunction with H_2_O_2_. As depicted in Fig. 2C, significant absorbance was recorded at λ = 446 nm when OPD substrate coexisted with H_2_O_2_ alongside MIL-101(Fe)-NH_2_. Correspondingly, visual observations indicated a color change from colorless to yellow within this solution system. Conversely, no color changes were noted in three control systems lacking catalyst involvement. When comparing signal peak intensities among samples, including both MIL-101(Fe)-NH_2_@MIP and its nonimprinted counterpart MIL-101(Fe)-NH_2_@NIP against pure MIL-101(Fe)-NH_2_, distinct variations were observed. These variations indicate differing degrees of peroxidase-like activities across these formulations, demonstrating that the peroxidase-mimic activities of MIL-101(Fe)-NH_2_@MIP and MIL-101(Fe)-NH_2_@NIP impaired owing to the coverage of imprinting layer. The molecularly imprinted cavities on the MIL-101(Fe)-NH_2_@MIP facilitate contact between H_2_O_2_ and MIL-101 (Fe)-NH_2_ within the imprinting layer, thereby still exhibit good peroxidase-mimic activity. Conversely, the absence of imprinted cavities in MIL-101(Fe)-NH_2_@NIP hinders interaction between external H_2_O_2_ and internal MIL-101(Fe)-NH₂, leading to a notable decrease in enzyme activity.

To evaluate the peroxide-like catalytic activity of both MIL-101(Fe)-NH_2_ and MIL-101(Fe)-NH_2_@MIP, steady-state kinetic tests were conducted using another chromogenic reagent, TMB, along with H_2_O_2_ as experimental substrates. A typical Michaelis-Menten curve was obtained by changing the concentration of either TMB or H_2_O_2_ while maintaining the other constant (Fig. 2C and D, Fig. S10). The hyperbolic fitting parameters-Michaelis-Menten constant (K_m_), maximal reaction velocity (V_max_) and catalytic constant (K_cat_) for both MIL-101(Fe)-NH_2_ and MIL-101(Fe)-NH_2_@MIP were calculated. As presented in Table S1, the calculated K_m_ value for MIL-101(Fe)-NH_2_ with TMB and H_2_O_2_ complex are 0.18 mM and 1.48 mM, respectively. When compared with HRP's K_m_ value toward TMB and H_2_O_2_ (0.43 mM and 3.7 mM, respectively), these results indicated that MIL-101(Fe)-NH_2_ exhibits superior catalytic performance relative to HRP. Conversely, for MIL-101(Fe)-NH_2_@MIP with TMB and H_2_O_2_ as substrates, K_m_ values are recorded at 0.27 mM and 2.34 mM, respectively. Additionally, the V_max_ and K_cat_ of MIL-101(Fe)-NH_2_@MIP is slower than that of MIL-101(Fe)-NH_2_, suggesting a reduced peroxide-like catalytic activity and catalytic efficiency because of the coverage of the imprinting layer. Nevertheless, MIL-101(Fe)-NH_2_@MIP still demonstrates commendable peroxidase-mimic activity when compared to HRP. To assess the long-term stability of the nanozyme's catalytic activity, we monitored its activity over an extended period. As shown in Fig. S11, MIL-101(Fe)-NH_2_@MIP exhibited remarkable stability, retaining 98 % of its initial activity after 30 days. This result indicates that the high stability of MIL-101(Fe)-NH_2_@MIP's catalytic performance.

The fluorescence spectrum of OPD incubated with various components were examined. As presented in Fig. 2F, MIL-101(Fe)-NH_2_@MIP exhibit a blue emission at 455 nm upon exciting at 380 nm. Upon introducing H_2_O_2_ and OPD, MIL-101(Fe)-NH_2_@MIP displays peroxidase-mimic activity by effectively oxidizing non-fluorescent OPD into yellow fluorescent DAP. Consequently, the emission signal at 455 nm decreases while a new emission peak attributed to DAP emerges at 560 nm. Similar phenomena are also observed for MIL-101(Fe)-NH_2_ (Fig. S12). To investigate the mechanism underlying this observation, we measured both the fluorescence spectrum of MIL-101(Fe)-NH_2_@MIP and the UV–vis absorption spectrum of DAP. As illustrated in Fig. S13, there is significantly overlap between the fluorescence emission spectrum (455 nm) of MIL-101(Fe)-NH_2_@MlP excited at 380 nm with the UV–vis absorption spectrum (446 nm) of DAP. Therefore, it can be concluded that the inhibition observed in the intrinsic fluorescence emission from MIL-101(Fe)-NH_2_@MIP is attributed to an internal filtration effect (IFE).

### 3.3 Feasibility analysis of CAP detection by MIL-101(Fe)-NH_2_@MIP

**Fig. 3:**
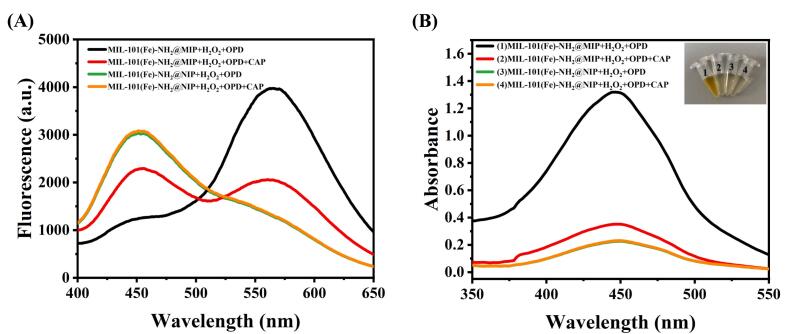
(A) fluorescence spectrum of various systems before and after the addition of CAP; (B) UV–vis spectrum of various systems before and after the addition of CAP.

The feasibility of employing MIL-101(Fe)-NH_2_@MIP for CAP determination was assessed through fluorescence spectra. As depicted in Fig. 3 A, following CAP addition to systems containing H_2_O_2_ and OPD due to MIP's high selectivity-the molecular imprinting cavity on MIL-101(Fe)-NH_2_@MIP specifically adsorbs CAP. This adsorption obstructs H_2_O_2_ from traversing through the imprinting layer and inhibits hydroxyl radical generation. Consequently, there is a reduction in fluorescent DAP production via hydroxyl radical oxidation from OPD. Ultimately, this results in a significant attenuation of DAP's fluorescence signal while simultaneously recovering the fluorescence signal from MIL-101(Fe)-NH_2_ (455 nm), owing to diminished IFE between them. Conversely, the MIL-101(Fe)-NH_2_@NIP, which lacks imprinted cavities, impedes the interaction between external H_2_O_2_ and internal MIL-101(Fe)-NH_2_, leading to significantly diminished enzyme activity. Given that the surface of MIL-101(Fe)-NH_2_@NIP does not possess memory cavities, there is a negligible effect on fluorescence intensity upon introducing CAP into the MIL-101(Fe)-NH_2_@NIP/ H_2_O_2_/OPD system. These findings demonstrate that the designed sensor is well-suited for fluorescence detection of CAP.

To further validate the feasibility of the developed MIL-101(Fe)-NH_2_@MIP sensing system, UV–vis absorption spectra were analyzed. As illustrated in Fig. 3B, since CAP possesses a matched size and shape compatible with the imprinted cavity, it can seal these channels, thereby inhibiting substrate infiltrate to the active site of MIL-101(Fe)-NH_2_@MIP. Consequently, this hinders the catalytic color reaction between OPD and H_2_O_2_. To substantiate this principle, comparisons were made between MIL-101(Fe)-NH_2_@MIP/H_2_O_2_/OPD system systems containing either CAP or no CAP. It was clearly observed that in absence of CAP, the system exhibited a dark yellow coloration. However, when CAP was introduced into the UV–visible absorption peak of MIL-101(Fe)-NH_2_@NIP/H_2_O_2_/OPD system remained largely unchanged. These results confirm that MIL-101(Fe)-NH_2_@MIP demonstrates good specificity for CAP and provide a foundation for its selective detection. Thus, a dual-channel sensing platform for detecting CAP has been established.

### 3.4 Experimental conditions optimization

To determine optimal conditions for detecting CAP, several parameters were optimized. Similar to peroxidase enzymes, it was found that the peroxidase-mimic activity of MIL-101(Fe)-NH_2_@MIP is pH-dependent. Therefore, experimental conditions were adjusted across different pH values to enhance its peroxidase-like activity. As shown in Fig. S14, at 446 nm wavelength within varying pH levels from 3.0 to 8.0 the absorption enhancement intensity of the MIL-101(Fe)-NH_2_@MIP/H_2_O_2_/OPD system displayed significant variation-reaching its maximum at pH 4.5. Secondly, the concentrations of H_2_O_2_ and OPD, as well as the reaction time for the catalytic oxidation of OPD by MIL-101(Fe)-NH_2_@MIP, were optimized. As illustrated in Figs. S15–17, the absorption enhancement intensity plateaued at 100 mM for H_2_O_2_, 1 mM for OPD, and a reaction time of 30 min. These conditions were thus chosen as optimal sensing parameters. Additionally, the recognition and detection of CAP are attributed to its interaction with MIL-101(Fe)-NH_2_@MIP. Consequently, both the molecular imprinting incubation time and dosage of MIL-101(Fe)-NH_2_@MIP were optimized. Fig. S18 demonstrates that the absorption enhancement value gradually increased with increasing doses of MIL-101(Fe)-NH_2_@MIP before stabilizing at a concentration of 10 μg/mL. As shown in Fig. S19, when the imprinted incubation time was set to 20 min, a plateau in reaction was achieved. Therefore, an optimal dose of 10 μg/mL for MIL-101(Fe)-NH_2_@MIP and an incubation period of 20 min for molecular imprinting were established.

### 3.5 Analysis performance and selectivity of MIL-101(Fe)-NH_2_@MIP for ratiometric fluorescent/colorimetric dual-mode detection of CAP

**Fig. 4:**
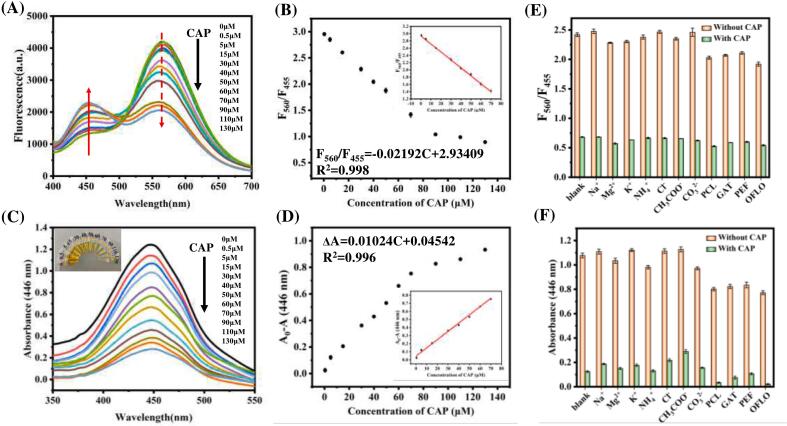
(A) shows the fluorescence profiles of MIL-101(Fe)-NH_2_@MIP/H_2_O_2_/OPD system in the presence of different CAP content; (B) describes the relationship between fluorescence intensity ration (F_560_/F_455_) and CAP content; (C) compares the effects of CAP and other species on response of MIL-101(Fe)-NH_2_@MIP/H_2_O_2_/OPD system; (D) The UV–visible absorption spectra of MIL-101(Fe)-NH_2_@MIP/H_2_O_2_/OPD system in the presence of different concentrations of CAP were shown; (E) the relationship between absorbance (446 nm) and CAP level was described; (F) The response of target CAP to MIL-101(Fe)-NH_2_@MIP/H_2_O_2_/OPD system was compared with that of other interferences.

Under the optimal experimental conditions described above, the developed fluorescence/colorimetric dual-mode sensor was employed for the quantitative detection of CAP. As illustrated in Fig. 4 A, an increase in CAP concentration resulted in a gradual enhancement of the intrinsic signal at 455 nm, while the peak fluorescent signal of DAP at 560 nm exhibited a corresponding decrease. Subsequently, fluorescence intensity values at both 455 nm and 560 nm were recorded. The fluorescence ratio F_560_/F_455_ was utilized as a metric to demonstrate the relationship between F_560_/F_455_ and CAP levels (Fig. 4B). The fluorescence ratio F_560_/F_455_ displayed a strong linear correlation with CAP concentrations spanning from 0.5 to 70 μM, yielding a fitting equation: F_560_/F_455_ = −0.02192 [CAP] + 2.93409 (R^2^ = 0.998). Based on a signal-to-noise ratio (S/N) of three, the limit of detection (LOD) for CAP was determined to be 36.45 nM. Additionally, absorbance responses from the MIL-101(Fe)-NH_2_@MIP/H_2_O_2_/OPD system to varying concentrations of CAP were also documented. As more CAP was introduced into the system, its yellow color gradually lightened and became discernible by eye. Correspondingly, with increasing amounts of CAP added, there was a progressive decline in UV–vis absorption signals (Fig. 4C). The decrease in absorbance at 446 nm demonstrated a robust linear relationship with CAP concentration within the range of 0.5–70 μM (Fig. 4D), providing a fitting equation for lower concentrations: A_446_-(A_446_)_0_ = 0.01024 [CAP] - 0.04542 (R^2^ = 0.996), with an LOD calculated at approximately 93.38 nM. In comparison to other methods for detecting CAP, our approach exhibits comparable or even superior performance regarding detection limits and sensing ranges as summarized in Table S2.

Another critical factor in assessing the performance of this assay is its capacity to resist interference from coexisting substances. Thus, selectivity tests were conducted prior to practical application. Competitive experiments involving CAP were carried out by measuring absorbance and fluorescence intensity ratio F_560_/F_455_ after introducing various interfering substances such as cation ions (Na^+^, K^+^, Mg^2+^, NH_4_^+^), anion ions (Cl^−^, CH_3_COO^−^, CO_3_^2−^) and other types of antibiotics (penicillin, gatifloxacin, pefloxacin, ofloxacin). As depicted in Figs. 4E and 4F, these interferences did not result in significant changes in fluorescence intensity ratios(F_560_/F_455_) or notable decreases in absorbance-even when their concentrations exceeded those of CAP by tenfold. This indicates that the developed dual-mode colorimetric and fluorescent assay exhibits excellent selectivity for CAP determination.

**Fig. 5:**
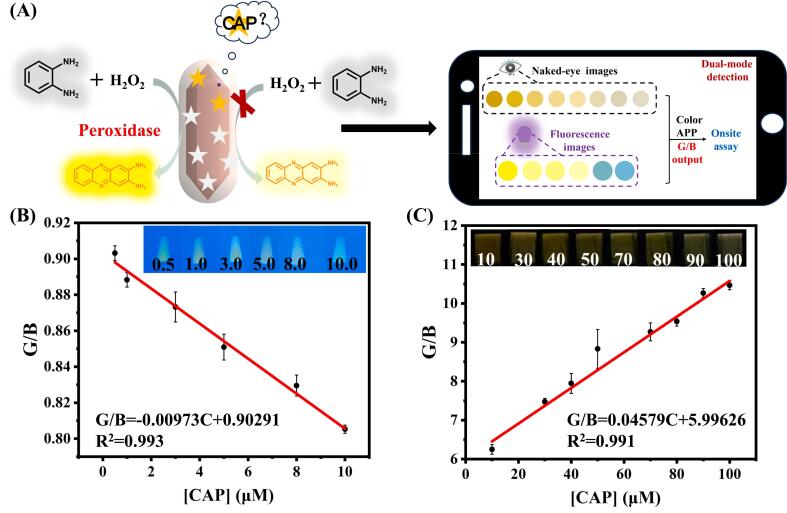
(A) Intelligent decision flow chart based on smart phone perception platform; (B) Relationship of the ratio of Green/Red channel values in fluorescent images and CAP concentrations varies from 0.5 to 10 μM; (C) Relationship of the ratio of Green/Red channel values in colorimetric images and CAP concentrations spans from 10 to 100 μM. (For interpretation of the references to color in this figure legend, the reader is referred to the web version of this article.)

The developed dual-mode fluorescent and colorimetric sensor was designed for practical applications by integrating it with a smartphone platform, enhancing its portability. Due to the ability to easily distinguish slight signal variations in color or fluorescence through naked-eye detection, a color image is captured using a smartphone and subsequently converted into the corresponding red (R), green (G), and blue (B) data via color picker software. Notably, Fig. 5B and C demonstrates a strong linear relationship between the ratio of green (G) to blue (B) channels and CAP concentration. The linear equation for fluorescence detection within the range of 0.5–10 μM is given by G/B = −0.00973 [CAP] + 0.90291 (R^2^ = 0.993), with a detection limit of 0.84 μM. In addition, the linear equation of colorimetric detection in the range of 10–100 μM is given by G/B = 0.04579 [CAP] + 5.99626 (R^2^ = 0.991), yielding a detection limit of 1.47 μM according to the 3σ rule. Collectively, the proposed sensor successfully achieved semi-quantitative detection through visual observation while providing quantitatively accurate measurements via smartphone integration, showcasing significant advantages and potential in CAP detection.

In order to evaluate the potential application of the fluorescence colorimetric dual-mode detection platform in real samples, recovery experiments for CAP were conducted using milk samples. Employing the standard addition method, various concentrations of CAP were introduced into these milk samples, and assays were performed according to the proposed detection protocol. As illustrated in Table 1, the recoveries obtained from both fluorescence and colorimetric methods ranged from 98.44 % to 100.28 % and 99.67 % to 100.64 %, respectively. Meanwhile, the relative standard deviations (RSD) varied between 1.06 % and 1.99 % for fluorescence and from 0.85 % to 3.47 % for colorimetry. These results confirm the high accuracy of our sensor in detecting CAP within real sample matrices.

**Table 1:** Analysis results of CAP in milk sample (*n* = 3).

Mode	Added (μM)	Found (μM)	Recovery (%)	RSD (n = 3)
Fluorescence	5.00	4.92	98.44	1.06 %
	10.00	9.95	99.46	1.99 %
	50.00	50.14	100.28	1.62 %
Colorimetric	5.00	4.98	99.67	1.52 %
	10.00	10.00	100.00	0.85 %
	50.00	50.32	100.64	3.47 %

## 4 Conclusion

In summary, MIL-101(Fe)-NH_2_@MIP was synthesized on the surface of MIL-101(Fe)-NH_2_ through a dopamine self-polymerization strategy, exhibiting excellent peroxidase-mimic activity along with specific recognition capabilities for chloramphenicol (CAP). By replacing expensive antibodies with the molecularly imprinted technology (MIT), MIL-101(Fe)-NH_2_@MIP probes enable ratiometric fluorescent and colorimetric dual-mode detection of CAP in aqueous solutions, while demonstrating exceptional selectivity and specificity for this antibiotic compound. This method's practical applicability has been validated through testing on real milk samples. Furthermore, the integrated MIL-101(Fe)-NH_2_@MIP smartphone sensing platform offers an on-site solution for fast and sensitive determination of CAP levels. Although the fabrication of the MIL-101(Fe)-NH_2_@MIP probe presents challenges in complexity, our dual-mode detection platform demonstrates analytical performance comparable to other CAP sensors while offering additional advantages: (i) it utilizes a single bifunctional MIL-101(Fe)-NH_2_@MIP probe that integrates enzyme-like activity with luminescent properties for dual-mode detection, enhancing reliability and reducing the likelihood of false positives. (ii) it streamlines the process by facilitating the simultaneous application of both detection methods under consistent experimental conditions to generate two independent response signals. (iii) dopamine's oxidative self-polymerization in alkaline solutions simplifies MIP synthesis, eliminating the need for functional monomers, crosslinkers, and harsh conditions. This work not only addresses the limitations of previous optical sensors but also paves the way for further exploration of nanozymes, presenting a promising and reliable strategy for antibiotic analysis.

## CRediT authorship contribution statement

**Xiang-Yi He:** Software, Investigation, Formal analysis, Data curation. **Ya Wang:** Visualization, Validation, Conceptualization. **Qin Xue:** Formal analysis, Data curation. **Wan-Fen Qian:** Software, Resources. **Guang-Li Li:** Validation, Resources, Formal analysis. **Qing Li:** Writing – review & editing, Writing – original draft, Supervision, Funding acquisition, Conceptualization.

## Declaration of competing interest

The authors declare that they have no known competing financial interests or personal relationships that could have appeared to influence the work reported in this paper.

## Data Availability

Data will be made available on request.

## Appendix A Supplementary data

Supplementary material Image 1
