# Supplementary material for: Molecularly imprinted MOF nanozymes: Demonstration of smartphone-integrated dual-mode platform for ratiometric fluorescent/colorimetric detection of chloramphenicol
Source: Food Chem X. 2025 Feb 25;26:102322. doi: 10.1016/j.fochx.2025.102322 (PMC11914186; doi:10.1016/j.fochx.2025.102322)
Supplement: Supplementary file 1 — Supplementary material [file mmc1.docx]

**Supporting Information**

**Molecularly imprinted MOF nanozymes: demonstration of smartphone-integrated dual-mode platform for ratiometric fluorescent/colorimetric detection of chloramphenicol**

Xiang-Yi He,^a^ Ya Wang,^b^ Qin Xue,^a^ Wan-Fen Qian,^a^ Guang-Li Li,^a^ Qing Li*^a^

*a. College of Life Science and Chemistry, Hunan University of Technology, Zhuzhou 412007, China.*

*b. Zhuzhou Institute for Food and Drug Control, Zhuzhou 412007, China.*

* Corresponding author.

E-mail address: qingli@hut.edu.cn

**Figure S1.** The EDS analysis of the prepared MIL-101(Fe)-NH_2_.

**Figure S2.** The UV-vis absorption spectra of supernatant after MIL-101(Fe)-NH_2_ was washed first and last time, respectively.

**Figure S3.** TEM images of MIL-101(Fe)-NH_2_@MIP.

**Figure S4.** The apparent zeta potential values of MIL-101(Fe)-NH_2_ and MIL-101(Fe)-NH_2_@MIP.

**Figure S5.** (A) Excitation (λex = 350 nm) and emission (λem = 450 nm) spectrum of the fluorescent MIL-101(Fe)-NH_2_. (B) Excitation (λex = 350 nm) and emission (λem = 450 nm) spectrum of the fluorescent NH_2_-BDC. (C) Fluorescence spectra of MIL-101(Fe)-NH_2_ at different excitation wavelengths.

**Figure S6.** (A) Excitation (λex = 350 nm) and emission (λem = 450 nm) spectrum of the fluorescent MIL-101(Fe)-NH_2_@MIP. (C) Fluorescence spectra of MIL-101(Fe)-NH_2_@MIP at different excitation wavelengths.

**Figure S7.** The effect of different concentration of salt solution on the fluorescence intensity of MIL-101(Fe)-NH_2_@MIP.

**Figure S8.** The effect of different temperature on the fluorescence intensity of MIL-101(Fe)-NH_2_@MIP.

**Figure S9.** (A) XPS analysis and the deconvolution of Fe 2p of MIL-101(Fe)-NH_2_.

**Figure S10.** Steady-state kinetic analyses using the Michaelis-Menten model and Lineweaver-Burk model for MIL-101(Fe)-NH_2_ by (A, B) varying the concentration of H_2_O_2_ with fixed amount of TMB and (C, D) varying the concentration of TMB with fixed amount of H_2_O_2_.

**Table S1.** Comparison of the kinetic parameters of HRP, MIL-101(Fe)-NH_2_ and MIL-101(Fe)-NH_2_@MIP.

| **Catalyst** | ***K*_m_(mM)** | | | ***V*_max_(10^-8^M/s)** | | ***Kcat* (S^-1^)** | | **Ref.** |
| --- | --- | --- | --- | --- | --- | --- | --- | --- |
|  | H_2_O_2_ | TMB | | H_2_O_2_ | TMB | H_2_O_2_ | TMB |  |
| HRP | 3.70 | 0.43 | 8.71 | | 10.00 | 3.48×10^3^ | 4.00×10^3^ | [1] |
| AuAg@PB_6_ | - | 0.17 | - | | 1029 | - | 1.46×10^7^ | [2] |
| PdCoO_x_-300 | 2.11 | 0.16 | 273220 | | 33320 | - | - | [3] |
| Ni-Pt NPs | - | 35 | - | | 260 | - | 4.50×10^7^ | [4] |
| MIL-101(Fe)-NH_2_ | 1.48 | 0.18 | 3.68 | | 9.14 | 3.68×10^8^ | 2.19×10^9^ | This work |
| MIL-101(Fe)-NH_2_@MIP | 2.34 | 0.27 | 2.47 | | 7.68 | 2.47×10^8^ | 1.84×10^9^ |  |

**Fig. S11** The effect of storage time on the peroxidase-like activity of MIL-101(Fe)-NH_2_@MIP.

**Figure S12.** Fluorescence spectra of different systems.

**Figure S13.** UV absorption spectra DAP and FL excitation and emission spectra of MIL-101(Fe)-NH_2_@MIP.

**Figure S14.** Optimization of pH value for peroxidase-mimicking activity of MIL-101(Fe)-NH_2_@MIP.

**Figure S15.** Optimization of H_2_O_2_ concentration for peroxidase-mimicking activity of MIL-101(Fe)-NH_2_@MIP.

**Figure S16.** Optimization of OPD concentration for peroxidase-mimicking activity of MIL-101(Fe)-NH_2_@MIP.

**Figure S17.** Optimization of reaction time for peroxidase-mimicking activity of MIL-101(Fe)-NH_2_@MIP.

**Figure S18.** Optimization of MIL-101(Fe)-NH_2_@MIP concentration for peroxidase-mimicking activity of MIL-101(Fe)-NH_2_@MIP.

**Figure S19.** Optimization of recognition time for peroxidase-mimicking activity of MIL-101(Fe)-NH_2_@MIP. **Table S2.** Comparison of previously reported for determination of CAP.

| **Material** | **Detection mode** | **Linear range (µM)** | | **LOD**  **(nM)** | **Ref.** |
| --- | --- | --- | --- | --- | --- |
| CuNCs | Fluorescence | 5-100 | 188.32 | | [5] |
| rGO/PdNPs | Electrochemistry | 50-1000 | 50 | | [6] |
| AuNPLs | Aptamer Sensor | 10-1000 | 500 | | [7] |
| MIP@SiO_2_@QDs | Fluorescence | 1-400 | 350 | | [8] |
| CD/MOFs | Fluorescence | 2-80 | 44 | | [9] |
| AgNWs | ELISA | 0.12-88.00 | 360 | | [10] |
| This work | Fluorescence | 0.50-70.00 | 36.45 | |  |
|  | Colorimetry |  | 93.38 | |  |

**References:**

[1] Gao L, Zhuang J, Nie L, Zhang J, Zhang Y, Gu N, Wang T, Feng J, Yang D, Perrett S, Yan X. Intrinsic peroxidase-like activity of ferromagnetic nanoparticles. Nat Nanotechnol. 2007, 2(9):577-83.

[2] Cai J, Lin Y, Yu X, Yang Y, Hu Y, Gao L, Xiao H, Du J, Wang H, Zhong X, Sun P, Liang X, Zhou H, Cai H. Multifunctional AuAg-doping prussian blue-based MOF: enhanced colorimetric catalytic activities and amplified SERS signals for bacteria discrimination and detection. Sensors and Actuators B: Chemical 2023, 394:134279.

[3] Wang L, Wang Y, Zhou Y. Bimetallic MOF-derived three-dimensional nanoflowers PdCoOx as peroxidase mimic activity for determining total antioxidant capacity. Food Chem. 2024, 457:140120.

[4] Xi Z, Wei K, Wang Q, Kim MJ, Sun S, Fung V, Xia X. Nickel-Platinum nanoparticles as peroxidase mimics with a record high catalytic efficiency. J Am Chem Soc. 2021, 143(7):2660-2664.

[5] Borse S, Murthy Z V P, Park T-J, Kailasa S K. Pepsin mediated synthesis of blue fluorescent copper nanoclusters for sensing of flutamide and chloramphenicol drugs. Microchemical Journal. 2021, 164:105947.

[6] Zhao H, Liu X, Cao Z, Zhan Y, Shi X, Yang Y, Zhou J, Xu J. Adsorption behavior and mechanism of chloramphenicols, sulfonamides, and non-antibiotic pharmaceuticals on multi-walled carbon nanotubes. Journal of Hazardous Materials. 2016, 310:235-245.

[7] Chang C-C, Wang G, Takarada T, Maeda M. Iodine-mediated etching of triangular gold nanoplates for colorimetric sensing of copper ion and aptasensing of chloramphenicol. ACS Applied Materials & Interfaces. 2017, 9(39):34518-34525.

[8] Chen X, Liu Y, Li P, Xing Y, Huang C. Molecularly imprinted silica-coated CdTe quantum dots for fluorometric determination of trace chloramphenicol. Molecules. 2021, 26(19):5965.

[9] Wang Q, Qi X, Chen H, Li J, Yang M, Liu J, Sun K, Li Z, Deng G. Fluorescence determination of chloramphenicol in milk powder using carbon dot decorated silver metal–organic frameworks. Microchimica Acta. 2022, 189(8):272.

[10] Kotelnikova P A, Iureva A M, Nikitin M P, Zvyagin A V, Deyev S M, Shipunova V O. Peroxidase-like activity of silver nanowires and its application for colorimetric detection of the antibiotic chloramphenicol. Talanta Open. 2022, 6:100164.
